# Effects and mechanisms of *Porphyromonas gingivalis* outer membrane vesicles induced cardiovascular injury

**DOI:** 10.1186/s12903-024-03886-7

**Published:** 2024-01-19

**Authors:** Jianbin Guo, Kaijin Lin, Siyi Wang, Xiaozhen He, Zhen Huang, Minqian Zheng

**Affiliations:** 1 Fujian Key Laboratory of Oral Diseases & Fujian Provincial Engineering Research Center of Oral Biomaterial & Stomatological Key Lab of Fujian College and University, School and Hospital of Stomatology, Fujian Medical University, Fuzhou, 350001 China; 2 Institute of Stomatology & Research Center of Dental and Craniofacial Implants, School and Hospital of Stomatology, Fujian Medical University, Fuzhou, 350001 China; 3 Institute of Life Sciences, College of Biological Science and Engineering, Fuzhou University, Fuzhou, 350108 China; 4 Innovation and Transformation Center, Fujian University of Traditional Chinese Medicine, Fuzhou, 350108 China; 5 College of Life Sciences, Fujian Normal University, Fuzhou, 350108 China

**Keywords:** Periodontitis, Cardiovascular disease, *Porphyromonas gingivalis*, Outer membrane vesicles, Transcriptome

## Abstract

**Background:**

The outer membrane vesicles (OMVs) derived from *Porphyromonas gingivalis* (*P. gingivalis*) have long been acknowledged for their crucial role in the initiation of periodontitis. However, the implications of *P. gingivalis* OMVs in the context of cardiovascular disease (CVD) remain incompletely understood. This study aimed to clarify both the impact and the underlying mechanisms through which *P*. *gingivalis* OMVs contribute to the propagation of distal cardiovascular inflammation and trauma.

**Methods:**

In this study, various concentrations (0, 1.25, 2.5, and 4.5 µg/µL) of *P. gingivalis* OMVs were microinjected into the common cardinal vein of zebrafish larvae at 48 h post-fertilization (hpf) to assess changes in cardiovascular injury and inflammatory response. Zebrafish larvae from both the PBS and the 2.5 µg/µL injection cohorts were harvested at 30 h post-injection (hpi) for transcriptional analysis. Real-time quantitative PCR (RT-qPCR) was employed to evaluate relative gene expression.

**Results:**

These findings demonstrated that *P. gingivalis* OMVs induced pericardial enlargement in zebrafish larvae, caused vascular damage, increased neutrophil counts, and activated inflammatory pathways. Transcriptomic analysis further revealed the involvement of the immune response and the extracellular matrix (ECM)-receptor interaction signaling pathway in this process.

**Conclusion:**

This study illuminated potential mechanisms through which *P. gingivalis* OMVs contribute to CVD. It accentuated their involvement in distal cardiovascular inflammation and emphasizes the need for further research to comprehensively grasp the connection between periodontitis and CVD.

**Supplementary Information:**

The online version contains supplementary material available at 10.1186/s12903-024-03886-7.

## Background

Periodontal diseases, as one of the most prevalent infection-induced inflammatory diseases, are inflammatory disorders affecting periodontal tissue, resulting in soft tissue recession, bone degradation, tooth loss, and a slight increase in systemic inflammatory markers [1, 2]. Extensive studies have illustrated the association between periodontitis and systemic diseases, particularly cardiovascular disease (CVD) [3, 4]. Central to this association is *Porphyromonas gingivalis* (*P. gingivalis*), a predominant pathogen in periodontitis and the development of CVD [5]. Toxins released by *P. gingivalis* can trigger a cascade of immune and inflammatory responses, releasing mediators into the bloodstream. This has the potential to accelerate atherosclerosis, worsen hypertension, impede myocardial infarction recovery, and consequently, heighten the risks and progression of CVD [6–8].

Recent studies have increasingly intrigued by how *P. gingivalis* contributes to cardiovascular harm [6, 9, 10]. Emerging evidence suggested that the outer membrane vesicles (OMVs) of bacterium might be instrumental in these adverse effects [10–12]. OMVs, spherical bilayer nanostructures abundant in Gram-negative bacteria, contain a myriad of biologically active materials. They house outer membrane proteins, lipopolysaccharides (LPS), phospholipids, nucleic acids, and some periplasm encapsulated during their formation, measuring between 20 and 250 nm in diameter [13]. Serving pivotal roles like supporting bacterial biofilm formation and delivering virulence factors to host cells, OMVs from *P. gingivalis* especially facilitate cell aggregation, aiding in plaque biofilm creation. These vesicles can not only transport active and concentrated molecules to distal sites, but also target specific sites due to the binding specificity between bacterial adhesins and receptors [13]. Hence, the release of *P. gingivalis* OMVs was suspected to play a central role in distal cardiovascular damage.

The zebrafish, a staple in biological research, shares a high gene conservation with humans [14]. Its transparent embryos, rapid development, and observable cardiovascular structure make it an exemplary model for studying CVD [15]. Fluorescent marker strains in zebrafish provide insights into the effects of *P. gingivalis* on CVD. Widziolek et al. infected zebrafish with genetically modified *P. gingivalis*, uncovering the first biological proof of the bacteria’s cardiovascular harm [16]. Subsequent investigations revealed the significant role of proteins on *P. gingivalis* OMVs, especially gingipain, in increasing cardiovascular cell permeability, triggering further damage [17]. However, the impact of *P. gingivalis* OMVs on CVD has not fully been understood, and this study aimed to uncover how *P*. gingivalis OMVs influence and trigger distal cardiovascular inflammation and trauma.

## Methods

### Ethics statement

The experimentation on animals was approved by the Scientific and Ethical Review Committee of Fujian Medical University, under the protocol IACUC FJMU 2023-Y-04103, in March 2023. All methodologies strictly conformed to the Animal Research: Reporting of In Vivo Experiments (ARRIVE) guidelines. Prior to euthanasia, zebrafish larvae were anesthetized with a 0.16 mg/ml Tricaine Methanesulfonate (MS222, Sigma A5040) solution. Euthanasia was then performed by immersing the zebrafish larvae in an ice-water mixture for 30 min.

### Culture of P. Gingivalis

Wild-type *P. gingivalis* strain ATCC 33,277 was inoculated into brain-heart infusion broth (Oxoid) containing 5 mg/mL yeast extract, 250 µg/mL L-cysteine, 1 mg/mL hemin, and 1 mg/mL vitamin K and incubated anaerobically (37 °C, 80% N_2_, 10% CO_2_, and 10% H_2_) [18, 19].

### Preparation, identification and observation of *P. Gingivalis* OMVs

For OMV isolation, freshly cultivated bacteria at an optical density of 600 (OD 600 = 1), which corresponded to 9 × 10^9^ colony-forming units (CFU), were subjected to centrifugation at 8,000 g and 4 °C for 5 min, resulting in the collection of particles. Filter the upper liquid (0.2 μm) and further centrifuge 1 h at 100,000 g at 4 °C [20]. OMVs were labeled with PKH26 (Bestbio, BB-441,125) for 10 min, and then the staining was stopped with an equal volume of 1% BSA. After isolation, OMV particles were washed, suspended in PBS, and subjected to analysis and characterization using a transmission electron microscope (TEM, Hitachi, HT-7700) and a nanoparticle size analyzer (NSA, NanoFCM, N30E). The protein concentration of OMVs was determined using the BCA protein concentration kit (TIANGEN, PA115) [21]. The yield of OMVs, expressed as the amount of protein (µg) obtained per 10^10^ bacteria, was calculated based on the bacterial count corresponding to OD 600 (5.6 × 10^10^ CFU/mL). Droplets containing OMVs were placed in a disk with a glass bottom (NEST, 801,001), and the images of OMVs were acquired on a Leica TCS-SP5 confocal microscope with an excitation light of 543 nm.

### Animal handling

Wild-type (WT) AB strain zebrafish, *Tg (fli1a: EGFP)* and *Tg (mpx:GFP)* (from the China Zebrafish Resource Center, Wuhan, China; http://zfish.cn) were maintained and raised according to the Zebrafish Book (Westerfield 2000). *Tg (*nfκb:*EGFP)* (from Fuzhou bio-service Biotechnology Co., Ltd. Fuzhou, China) was constructed following the report [12].

### Treatment of zebrafish with *P. Gingivalis* OMVs for injury test

A minimum of 5 zebrafish larvae per group was mandated, with each group being replicated thrice, amounting to a total of 15 specimens. At 30 h post-injection (hpf), zebrafish embryos were dechorionated with help of sharp-tipped forceps and anesthetized with 0.04 mg/ml of tricaine (MS-222, Sigma). Anesthetized embryos were transferred onto a modified agarose gel for microinjection. Approximately 4 nL of PBS or OMVs (0, 1.25, 2.5 and 4.5 µg/µL) were injected into the common cardinal vein [8, 10] using an Eppendorf microinjector (FemtoJet 4i, Eppendorf). PBS injected group was used as a control group. The experimental unit was per zebrafish larvae. After microinjection, the zebrafish larvae were anesthetized and observed with the confocal microscope. At 2 hpi, 24 hpi and 48 hpi, live imaging was performed under a stereoscopic fluorescence microscope (SMZ800N). The fluorescence density was evaluated with the ImageJ software, and data from 15 zebrafish larvae in each group were counted. Briefly: ImageJ - File - image - Type − 8bit - Edit - inverse - Analyze - calibrate - Function - uncalibrated OD - Global calibration - Set Scale - click to Remove Scale - Set Measurements - Area - Image - Adjust - Threshold - Analyze - Measure; The unit is pixel; Mann Whitney test was used to analyze the significant difference between groups (*p < 0.05, **p < 0.01, ***p < 0.001, ****p < 0.0001).

### mRNA sequencing and data analysis

Zebrafish larvae were treated with *P. gingivalis* OMVs as mentioned above. Zebrafish larvae from the PBS and 2.5 ng/L injection groups at 48 hpi were collected and used for transcriptional profiling. 30 zebrafish larvae were pooled as one composite sample (*n* = 3). Then, the samples were sent to do mRNA sequencing for transcriptional profiling (Novogene, China). Briefly, mRNA was purified from total RNA using poly-Tol-igo-attached magnetic beads. RNA integrity was assessed by the RNA Nano 6000 Assay Kit of the Bioanalyzer 2100 system (Agilent Technologies, CA, USA). The extracted RNA was reversely transcribed, and the library was built according to the standard process. The library quality was assessed on the Agilent Bioanalyzer 2100 system and the library preparations were sequenced on an Illumina Novaseq platform and 150 bp paired-end reads were generated. Clean data (clean reads) were obtained by removing reads containing adapter, reads containing ploy-N and low-quality reads from raw data. At the same time, Q20, Q30 and GC content the clean data were calculated. The clean reads were then aligned with the genome sequence reference (GRCz11 release100). Index of the reference genome was built using Hisat2 v2.0.5 and paired-end clean reads were aligned to the reference genome using Hisat2 v2.0.5. The mapped reads of each sample were assembled by StringTie (v1.3.3b) in a reference-based approach [22]. FeatureCounts v1.5.0-p3 was used to count the reads numbers mapped to each gene. Differential expression analysis of two conditions/groups (two biological replicates per condition) was performed using the DESeq2 R package (1.20.0). The resulting P-value were adjusted in the Benjamini and Hochberg’s approach for controlling the false discovery rate. Genes with an adjusted *P* value < 0.05 found by DESeq2 were assigned as differentially expressed.

Gene ontology (GO) enrichment analysis of differentially expressed genes was implemented by the cluster Profiler R package, in which gene length bias was corrected. GO terms with a corrected *P* value less than 0.05 were considered significantly enriched by differential expressed genes. Kyoto Encyclopedia of Genes and Genomes (KEGG) is a database resource for understanding high-level functions and utilities of the biological system, such as the cell, the organism and the ecosystem, from molecular-level information, especially large-scale molecular datasets generated by genome sequencing and other high-through put experimental technologies (http://www.genome.jp/kegg/). Cluster Profiler R package was used to test the statistical enrichment of differential expression genes in KEGG pathways.

Read data had been uploaded to the NCBI Sequence Read Archive database (Ac-cession no.: SRR26319642, SRR26316552, SRR26298124, SRR26283523, SRR26284600, and SRR26213372).

### RNA isolation and real-time quantitative PCR (RT-qPCR)

RNAiso Plus (TakaRa) was employed to extract total RNA from the PBS and 2.5 ng/L injection groups at 48 hpi and then reversely transcribed to cDNA using Novo-Script® 1st Strand cDNA Synthesis SuperMix (Novoprotein) [23]. Subsequently, RT-qPCR was conducted on an ABI PRISM 7500 (Applied Biosystems, USA) with SYBR® Premix Ex TaqTM kit (Takara). ArchimedTM X4 (ROCGENE, Jiangshu, China) and NovoStart® SYBR qPCR SuperMix Plus were utilized for RT-qPCR (Novoprotein). All measures were conducted according to the manufacturer’s instructions. The primers for *NK2 homeobox 5* (*nkx2.5*), *GATA binding protein 4* (*gata4*), *cadherin 5* (*cdh5*), *tumor necrosis factor a* (*tfna*), *tumor necrosis factor b* (*tnfb*), *interleukin 6* (*Il6*), *laminin subunit alpha 3* (*lama3)*, *laminin subunit beta 3* (*lamb3)*, *laminin subunit gamma 2* (*lamc2)*, *synaptic vesicle glycoprotein 2 A* (*sv2a)* and *thrombospondin 1a* (*thbs1a)* were listed in Table S1. The relative change of mRNA levels was calculated by the 2 − ΔΔCt method and β-actin was used as the reference [24]. A t-test and one-way analysis.

of variance (ANOVA) with Dunnett’s multiple comparison tests were applied to calculate the significance in qPCR data by the GraphPad Prism 9 software (GraphPad Software, San Diego California, USA). Data were shown as the mean ± standard deviation (SD), and *P* values < 0.05 were considered statistically significant. Biological triplicates and three method repetitions were performed on each sample.

## Results

### Characterization of *P. Gingivalis* OMVs

**Fig. 1:**
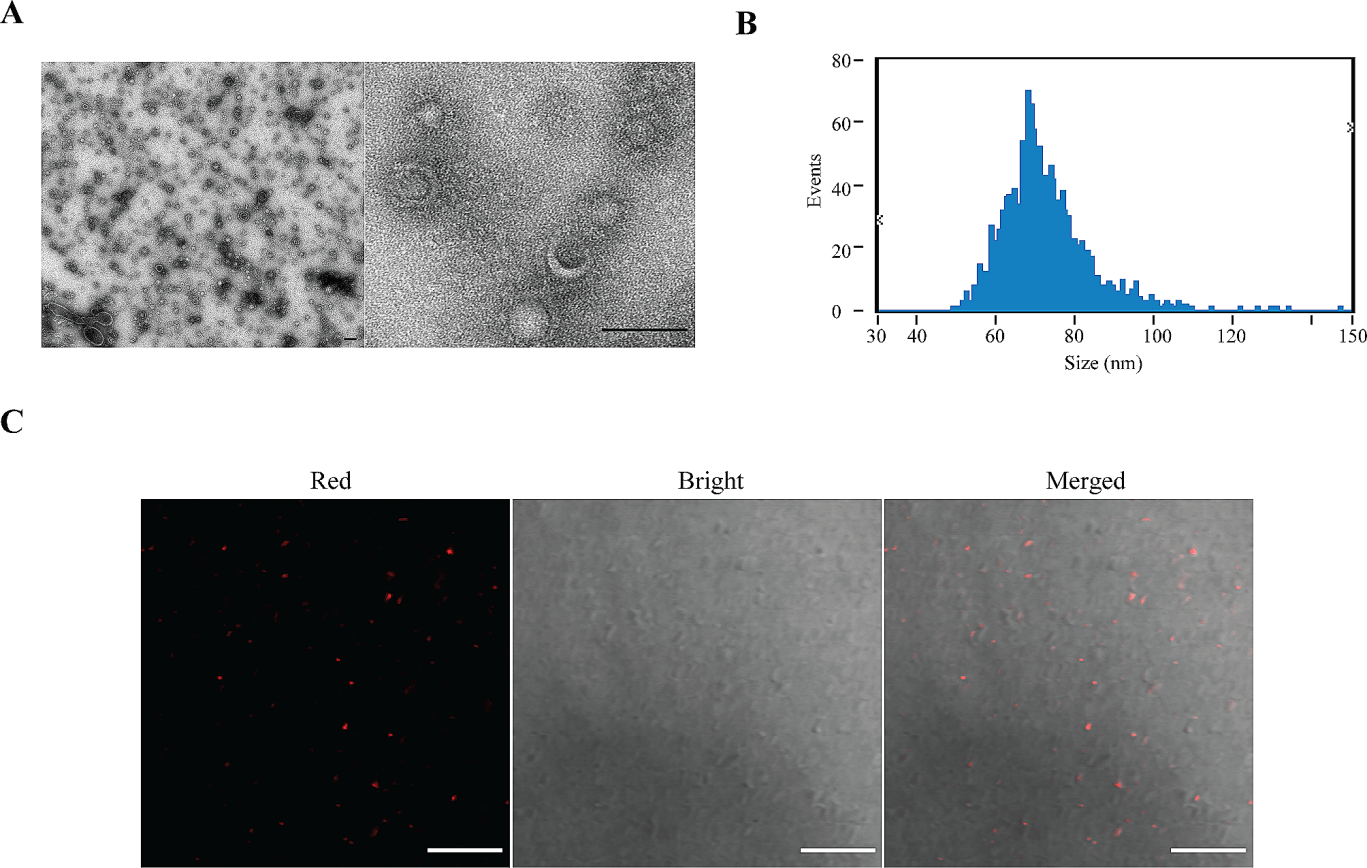
Characterization of *P. gingivalis* outer membrane vesicles (OMVs). (**A**) Transmission electron microscope (TEM) micrographs showing *P. gingivalis* OMVs. Electron microscopy imaging at 100 kv. Scale bar: 100 nm. (**B**) Size characteristics of *P. gingivalis* OMVs with a nanoparticle size analyzer (NSA). The dilution factor is 1000. The average diameter of *P. gingivalis* OMVs was about 72.28 nm with a concentration of 8.19 × 10^10^ particles/mL. (**C**) Confocal imaging results of *P. gingivalis* OMVs under 63x oil lens. Red, *P. gingivalis* OMVs labeled with PKH26. Scale bar: 10 μm

In order to explore the impact of *P. gingivalis* OMVs on cardiovascular injury, *P. gingivalis* OMVs were extracted from the *P. gingivalis* by low-temperature transcendation, and identified through NSA and TEM. A typical round vesicle-like morphology was observed (Fig. 1A) with an average diameter of about 72.28 nm, consistent with previous research reports (Fig. 1B) [12, 15]. For experimental convenience, all OMVs were labeled with PKH26, detected with confocal imaging and quantified according to protein content (Fig. 1C).

### OMVs of *P. gingivalis* caused cardiac injury to zebrafish larvae

**Fig. 2:**
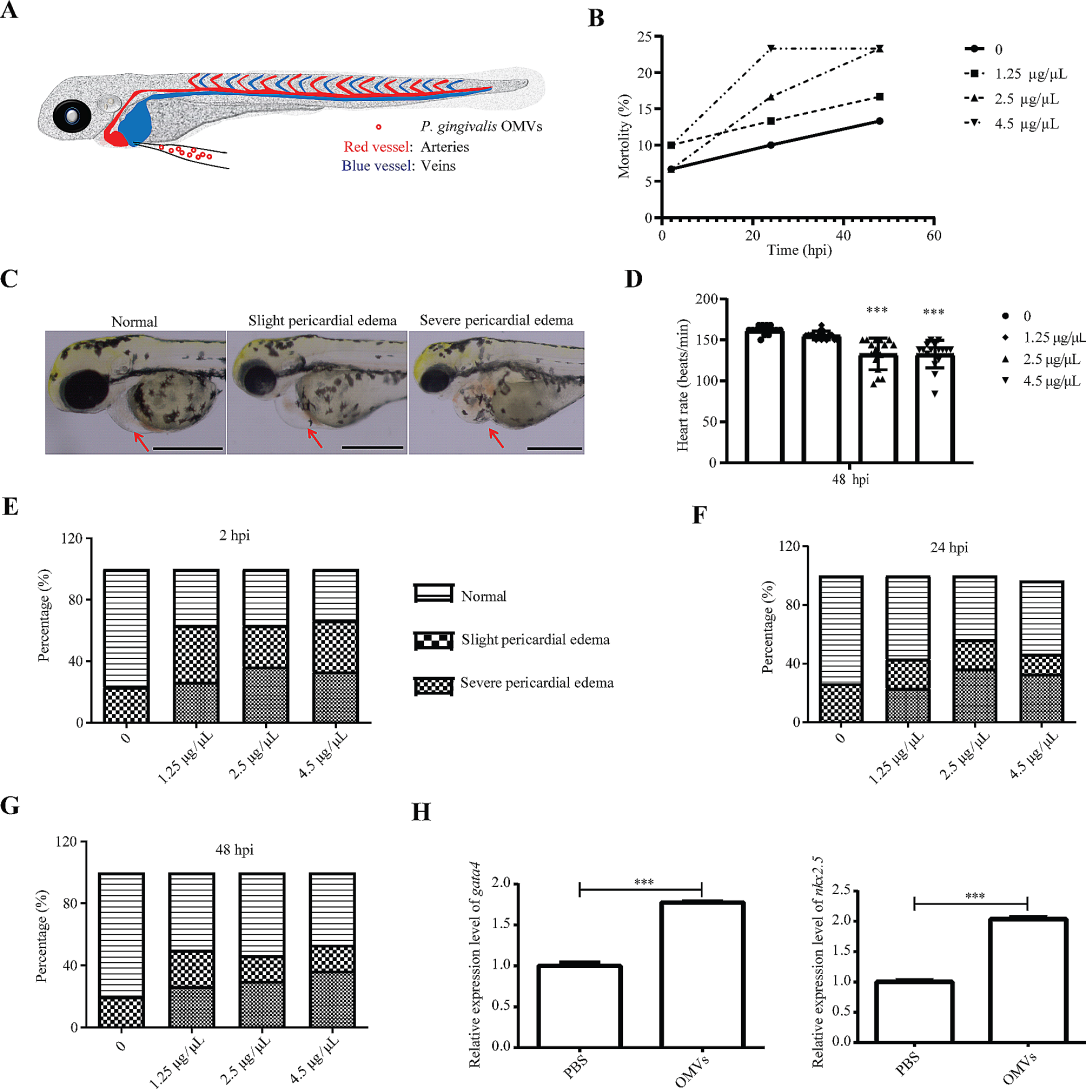
Analysis of the impact of *P. gingivalis* OMVs on the cardiac system. (**A**) Schematic diagram of the operation of the *P. gingivalis* OMVs for zebrafish treatment. Approximately 4 nL of PBS or OMVs were injected into the common cardinal vein of zebrafish larvae at 30 hpf using an Eppendorf microinjector (FemtoJet 4i, Eppendorf). (**B**) Mortality of zebrafish treated with *P. gingivalis* OMVs with different concentrations (0, 1.25, 2.5 and 4.5 µg/µL) at 2, 24 and 48 h post injection (hpi). *n* = 30, each group being replicated thrice. (**C**) Representative diagram of cardiac phenotype of zebrafish larvae at 48 hpi. Red arrow indicates pericardial position. The zebrafish larvae treated with OMVs showed three degrees of pericardial edema, that is normal, slight pericardial edema and severe pericardial edema. Scale bar: 0.5 mm. (**D**) Heartbeat statistics for different treatment groups (0, 1.25, 2.5 and 4.5 µg/µL) at 48 hpi. The unit is beats/min. *n* = 15, each group being replicated thrice. ***p ≤ 0.001. (**E**) Percentage of pericardial edema at concentrations of 0, 1.25, 2.5 and 4.5 µg/µL in 2 hpi. *n* = 30, each group being replicated thrice. (**F**) Percentage of pericardial edema at concentrations of 0, 1.25, 2.5 and 4.5 µg/µL in 24 hpi. *n* = 30, each group being replicated thrice. (**G**) Percentage of pericardial edema at concentrations of 0, 1.25, 2.5 and 4.5 µg/µL in 48 hpi. *n* = 30, each group being replicated thrice. (**H**) Expression results of *gata4* and *nkx 2.5* genes in 48 hpi zebrafish larvae. The relative change of mRNA levels was calculated by the 2 − ΔΔCt method and β-actin. Data were shown as the mean ± standard deviation (SD), and *P* values < 0.05 were considered statistically significant, ***p ≤ 0.001

To test the damaging effects of *P. gingivalis* OMVs on heart, *P. gingivalis* OMVs was prepared into three concentrations: 1.25 µg/µL, 2.5 µg/µL and 4.5 µg/µL through protein content. About 4 nL *P. gingivalis* OMVs were injected into the blood circulation of zebrafish larvae at 30 hpi by microinjection technology to observe the changes of relevant cardiac phenotypes (Fig. 2A). The control group was injected with the same volume of PBS. The mortality, heart rate, the proportion of pericardium enlargement and the proportion of zebrafish larvae with abnormal blood flow were counted at 2 hpi, 24 hpi and 48 hpi. The results demonstrated that the mortality of *P. gingivalis* OMVs treat groups were comparable to that of PBS group at 2 hpi (Fig. 2B). However, the mortality of *P. gingivalis* OMVs treatment rose with the increase of concentration at 24 hpi, and the mortality rate of 4.5 µg/µL group at 24 hpi was 23.33% (Fig. 2B). Over time, the mortality at 24 hpi and 48 hpi remained basically stable (Fig. 2B). The zebrafish larvae treated with OMVs showed three degrees of pericardial edema: normal, slight pericardial edema and severe pericardial edema (Fig. 2C). Furthermore, the heartbeat rates slowed significantly in the 2.5 µg/µL and 4.5 µg/µL *P. gingivalis* OMV treat groups at 24 hpi and 48 hpi (Fig. 2D). In addition, by randomly counting the live zebrafish larvae, it was found that more than 60% of the zebrafish larvae treated with different concentrations of *P. gingivalis* OMV had pericardial edema at 2 hpi (Fig. 2E). At 24 hpi, the proportions of severe pericardial edema were 23.33%, 36.67% and 33.33% in 1.25 µg/µL, 2.5 µg/µL and 4.5 µg/µL groups, respectively (Fig. 2F). The malformation rate of 48 hpi was similar to that of 24 hpi (Fig. 2G). Therefore, based on the experimental results, we decided to select 2.5 µg/µL concentration for subsequent experiments. In order to further reveal the impact of *P. gingivalis* OMVs on the cardiac system, we detected the genes related to the cardiac system by RT-qPCR. The results showed that there were significant differences in the expression of *gata4* and *nkx2.5* between 2.5 µg/µL *P. gingivalis* OMVs group and PBS group at 48 hpi (Fig. 2H). The expression of these genes was significantly upregulated in the *P. gingivalis* OMVs group, further indicating that the cardiac system was impaired in *P. gingivalis* OMVs treated zebrafish. Through this part of the study, it was clear that the OMVs of periodontal pathogen *P. gingivalis* could increase cardiac damage response such as pericardial enlargement of zebrafish.

### OMVs of *P. gingivalis* caused blood vessel injury to zebrafish

**Fig. 3:**
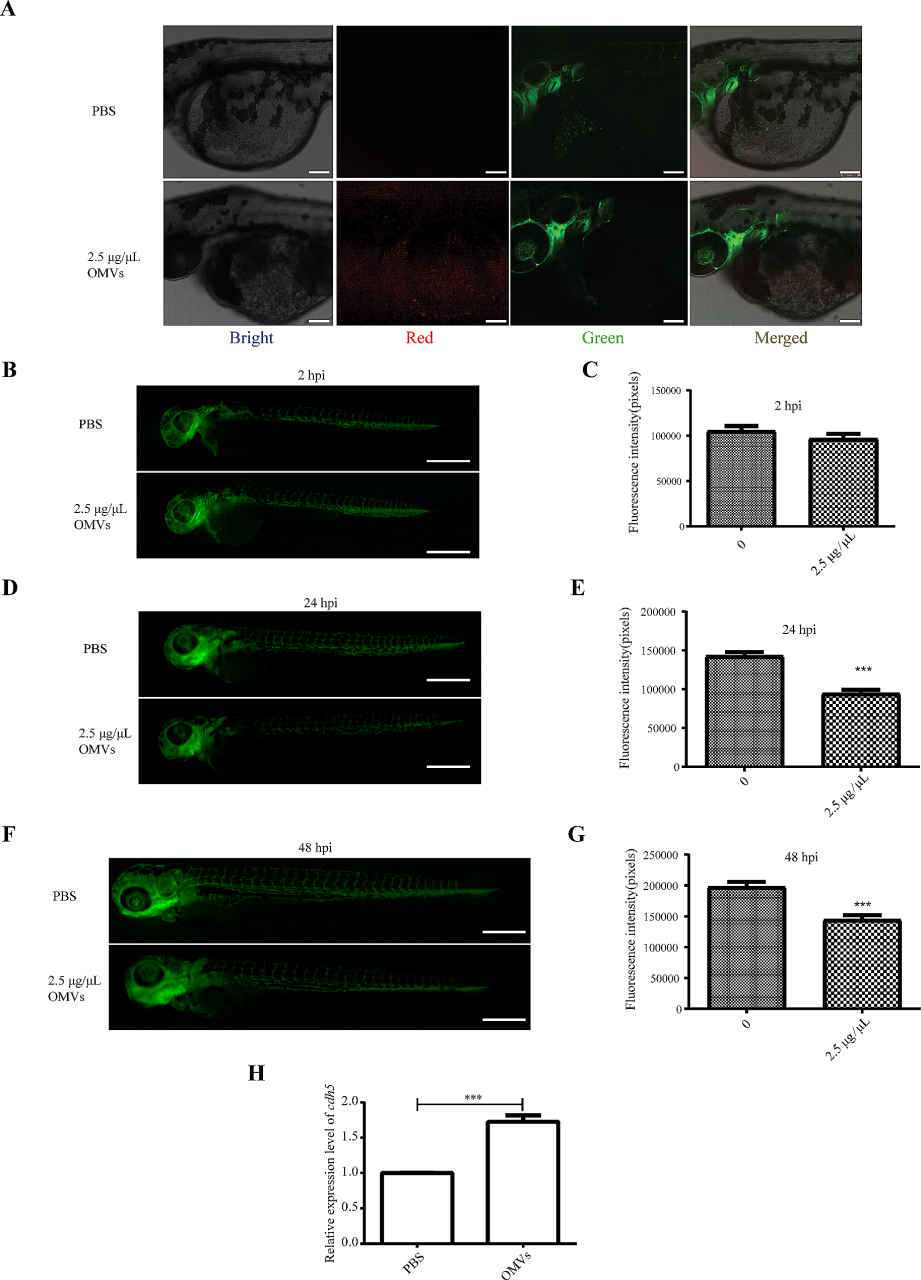
Analysis of the impact of *P. gingivalis* OMVs on the vascular system. (**A**) Representation of confocal image of zebrafish larver at 0 hpi with microinjection of PBS or 2.5 µg/µL *P. gingivalis* OMVs in that heart region of the body. Bright, Bright channel, Red, *P. gingivalis* OMVs; Green, vascular endothelial cells. Scale bar: 100 μm. (**B**) Image of vascular fluorescence at 2 hpi after treated with PBS or 2.5 µg/µL *P. gingivalis* OMVs. Live imaging was performed under a stereoscopic fluorescence microscope (SMZ800N) in the green channel. Scale bar: 0.5 mm. (**C**) Analysis results of vascular fluorescence according to picture taken in Fig. 3B. *n* = 15, each group being replicated thrice. The unit is pixel, Mann Whitney test was used to analyze the significant difference between groups. (**D**) Image of vascular fluorescence at 24 hpi after treated with PBS or 2.5 µg/µL *P. gingivalis* OMVs. Scale bar: 0.5 mm. (**E**) Analysis results of vascular fluorescence according to picture taken in Fig. 3D. *n* = 15, each group being replicated thrice. Mann Whitney test was used to analyze the significant difference between groups, ***p ≤ 0.001. (**F**) Image of vascular fluorescence at 48 hpi after treated with PBS or 2.5 µg/µL *P. gingivalis* OMVs. Scale bar: 0.5 mm. (**G**) Analysis results of vascular fluorescence according to picture taken in Fig. 3F. *n* = 15, each group being replicated thrice. Mann Whitney test was used to analyze the significant difference between groups, ***p ≤ 0.001. (**H**) Expression results of *cdh5* genes in 48 hpi zebrafish larvae. The relative change of mRNA levels was calculated by the 2 − ΔΔCt method and β-actin. Data were shown as the mean ± standard deviation (SD), and *P* values < 0.05 were considered statistically significant, ***p ≤ 0.001

To further test the damaging effects of *P. gingivalis* OMVs on blood vessels, a visualization system was developed with *Tg (fli1a: EGFP)* zebrafish line in which vascular endothelial cells were displayed with a fluorescent protein under the promoter of *fli1a*. Embryos of *Tg (fli1a: EGFP)* line fish were collected for microinjection, and 2.5 µg/µL PKH26 labeled *P. gingivalis* OMVs were injected into 30 hpf zebrafish larvae and images were collected by confocal fluorescence microscope or stereomicroscopy with PBS as a control. PKH26 labeled *P. gingivalis* OMVs could be imaged by confocal imaging, revealing that it has been successfully injected into zebrafish larvae (Fig. 3A). Statistical analysis revealed that there was no significant difference among the 2 hpi treatment groups, while the mean fluorescence density of the 24 hpi and 48 hpi zebrafish larvae *P. gingivalis* OMVs injection groups was significantly weaker than that of the PBS control group, indicating that the blood vessels of zebrafish were damaged by *P. gingivalis* OMVs (Fig. 3B-G). In addition, the expression of *cdh5*, a gene related to vascular development and functional maintenance, was significantly increased in the OMVs treated group (Fig. 3H).

### OMVs of *P. Gingivalis* triggered inflammatory reactions to zebrafish

**Fig. 4:**
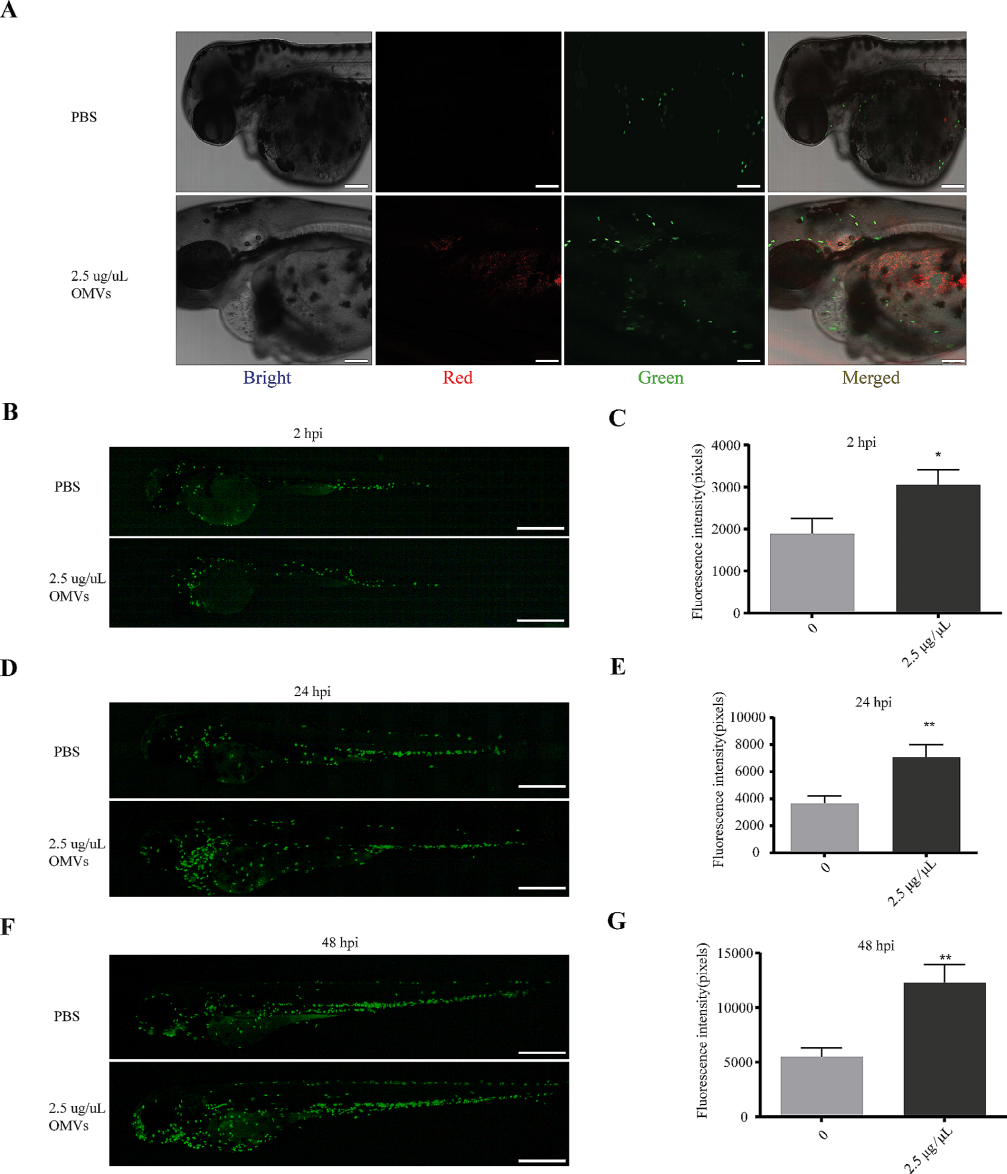
Analysis of the impact of *P. gingivalis* OMVs on the neutrophils. (**A**) Representation of confocal image at 0 hpi with microinjection of PBS or 2.5 µg/µL *P. gingivalis* OMVs in that heart region of the body. Bright, Bright channel, Red, *P. gingivalis* OMVs; Green, neutrophils. Scale bar: 100 μm. (**B**) Image of neutrophils fluorescence at 2 hpi after treated with PBS or 2.5 µg/µL *P. gingivalis* OMVs. Live imaging was performed under a stereoscopic fluorescence microscope (SMZ800N) in the green channel. Scale bar: 0.5 mm. (**C**) Analysis results of neutrophils fluorescence according to picture taken in Fig. 4B. *n* = 15, each group being replicated thrice. The unit is pixel, Mann Whitney test was used to analyze the significant difference between groups. *p < 0.05. (**D**) Image of neutrophils fluorescence at 24 hpi after treated with PBS or 2.5 µg/µL *P. gingivalis* OMVs. Scale bar: 0.5 mm. (**E**) Analysis results of neutrophils fluorescence according to picture taken in Fig. 4D. *n* = 15, each group being replicated thrice. Mann Whitney test was used to analyze the significant difference between groups, **p < 0.01. (**F**) Image of neutrophils fluorescence at 48 hpi after treated with PBS or 2.5 µg/µL *P. gingivalis* OMVs. Scale bar: 0.5 mm. (**G**) Analysis results of neutrophils fluorescence according to picture taken in Fig. 4F. *n* = 15, each group being replicated thrice. Mann Whitney test was used to analyze the significant difference between groups, **p < 0.01

In order to further investigate the effects of *P. gingivalis* OMVs on zebrafish larvae, *Tg (mpx: EGFP)*, a zebrafish strain marked neutrophils with green fluorescent protein (GFP) was adopted. Similarly, by confocal microscopy, the red fluorescent labeled *P. gingivalis* OMV was observed in the larve fish (Fig. 4A). At 2 hpi, the number of neutrophils in the *P. gingivalis* OMV treated group was significantly higher than that in the PBS group, and this situation did not improve at 24 hpi and 48 hpi (Fig. 4B-G).

**Fig. 5:**
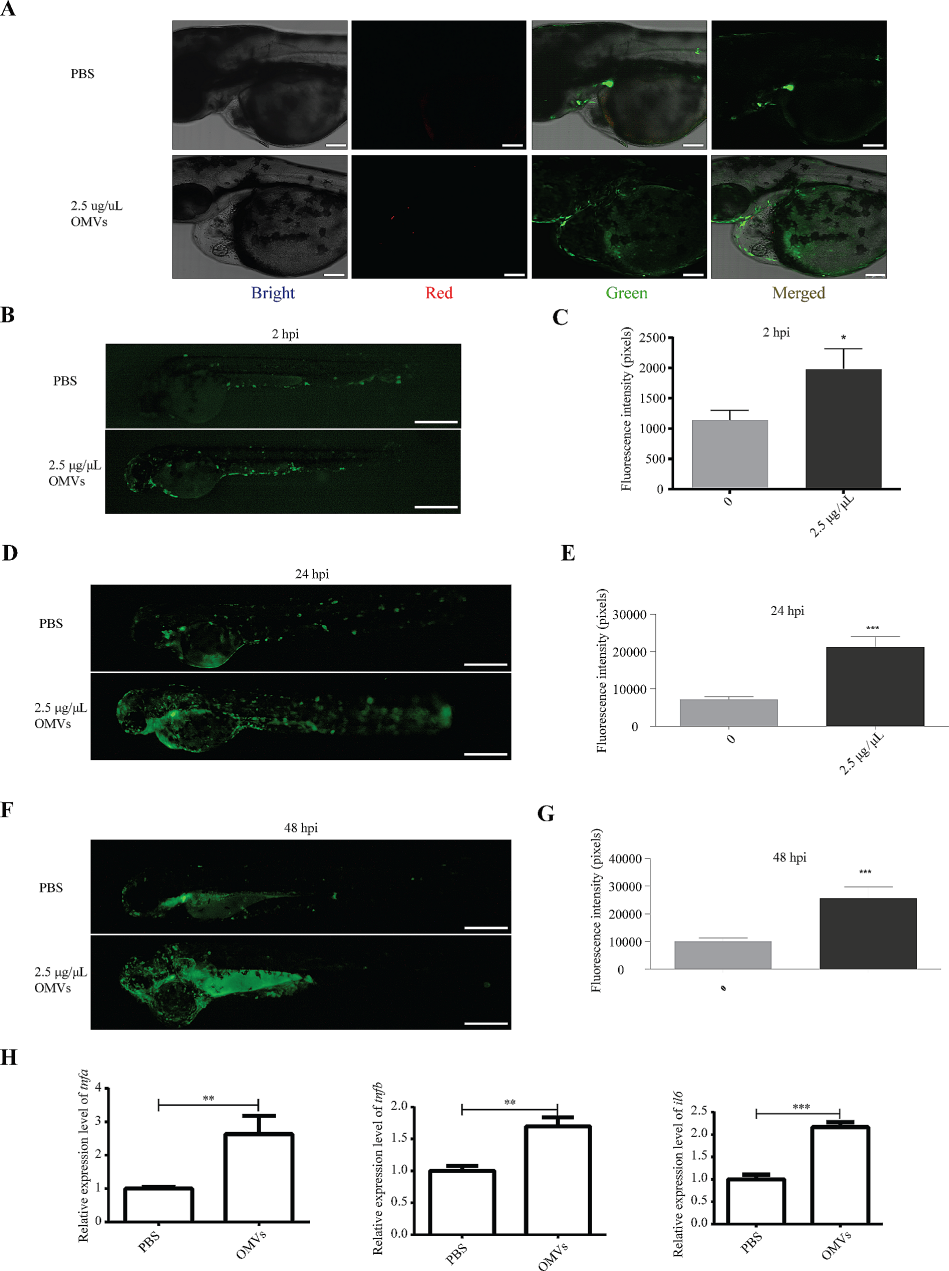
Analysis of the impact of *P. gingivalis* OMVs on the nfκb signaling pathway. (**A**) Representation of confocal image at 0 hpi with microinjection of PBS or 2.5 µg/µL *P. gingivalis* OMVs in that heart region of the body. Bright, Bright channel, Red, *P. gingivalis* OMVs; Green, nfκb signaling. Scale bar: 100 μm. (**B**) Image of green fluorescence at 2 hpi after treated with PBS or 2.5 µg/µL *P. gingivalis* OMVs. Live imaging was performed under a stereoscopic fluorescence microscope (SMZ800N) in the green channel. Scale bar: 0.5 mm. (**C**) Analysis results of green fluorescence according to picture taken in Fig. 5B. *n* = 15, each group being replicated thrice. The unit is pixel, Mann Whitney test was used to analyze the significant difference between groups. *p < 0.05. (**D**) Image of green fluorescence at 24 hpi after treated with PBS or 2.5 µg/µL *P. gingivalis* OMVs. Scale bar: 0.5 mm. (**E**) Analysis results of green fluorescence according to picture taken in Fig. 5D. *n* = 15, each group being replicated thrice. Mann Whitney test was used to analyze the significant difference between groups, ***p ≤ 0.001. (**F**) Image of green fluorescence at 48 hpi after treated with PBS or 2.5 µg/µL *P. gingivalis* OMVs. Scale bar: 0.5 mm. (**G**) Analysis results of green fluorescence according to picture taken in Fig. 5F. *n* = 15, each group being replicated thrice. Mann Whitney test was used to analyze the significant difference between groups, ***p ≤ 0.001. (**H**) Expression results of *tnfa, tnfb* and *il6* genes in 48 hpi zebrafish larvae. The relative change of mRNA levels was calculated by the 2 − ΔΔCt method and β-actin. Data were shown as the mean ± standard deviation (SD), and *P* values < 0.05 were considered statistically significant, **p < 0.01, ***p ≤ 0.001

Moreover, to further clarify whether the cardiovascular injury induced by *P. gingivalis* OMVs involves the nfκb signaling pathway, 2.5 µg/µL *P. gingivalis* OMVs were injected into *Tg (nfκb: EGFP)* zebrafish larvae (Fig. 5A). The *Tg (nfκb: EGFP)* is a line in which GFP was driven by the promoter of nfκb gene, which is able to respond to inflammation and activate the expression of GFP. It was indicated that the nfκb signaling pathway in zebrafish larvae injected with *P. gingivalis* OMVs was significantly enhanced at 2 hpi, and that the difference was more obvious at 24 hpi and 48 hpi (Fig. 5B-G). Inflammatory-related genes, such as *tnfa*, *tnfb* and *il6*, were also tested through RT-qPCR, and it was found that these genes had increased expressions from 1.7 to 2.6 fold (Fig. 5H). These results revealed that the nfκb signaling pathway may be involved in the effects of *P. gingivalis* OMVs on cardiovascular damage.

### Transcriptomic changes in *P. Gingivalis* OMVs treated zebrafish

**Fig. 6:**
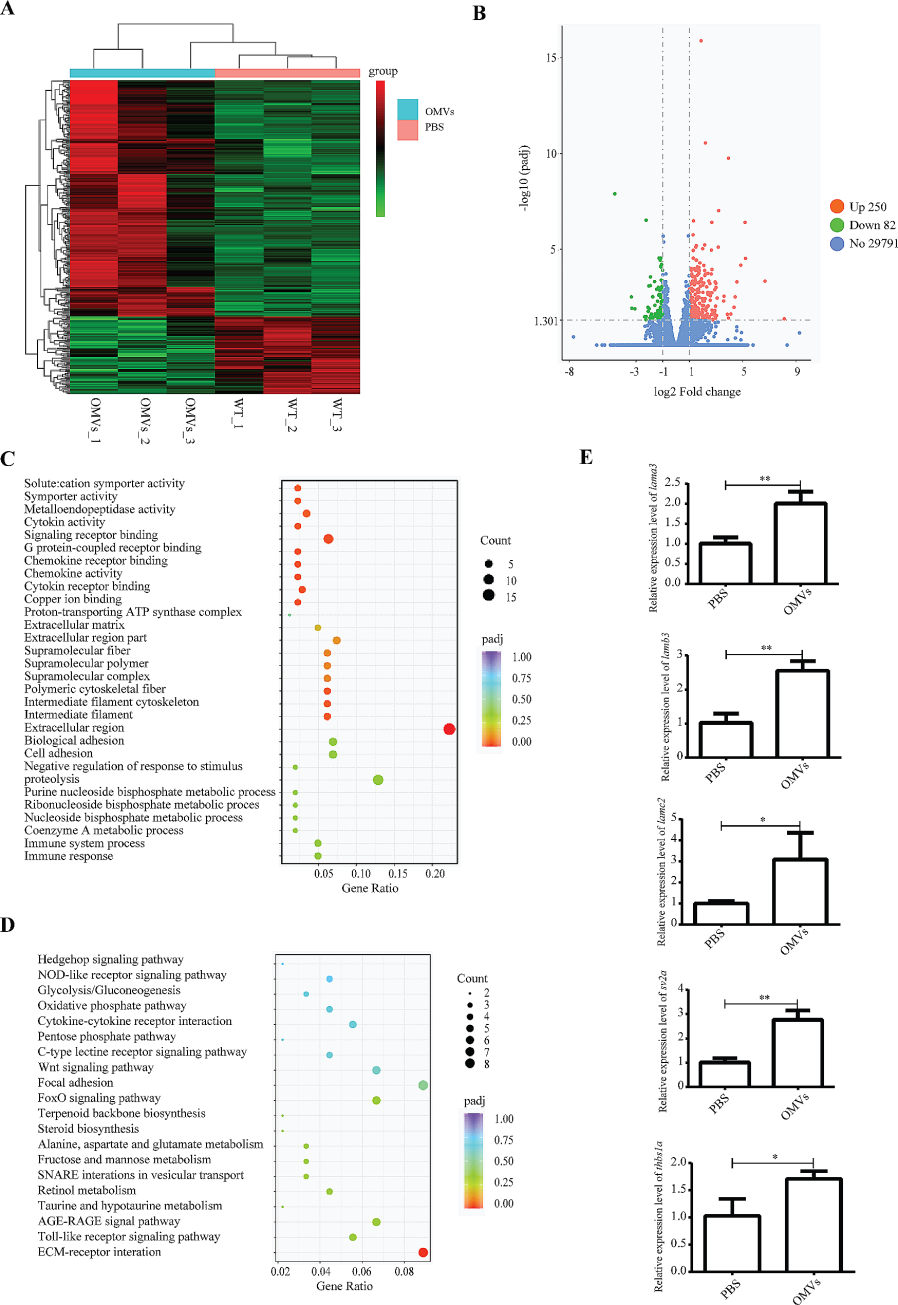
Transcriptomics results and validation. (**A**) Heat map analysis for RNA-seq data of samples at 48 hpi from the zebrafish larvae injected with PBS and *P. gingivalis* OMVs. (**B**) Volcano plot of differential expression analysis of PBS and *P. gingivalis* OMVs treated zebrafish larvae showing the relationship between P-value and log fold changes. Red shows upregulated genes and blue downregulated genes. Relative to the control, 332 differentially expressed genes (DEGs), including 250 up-regulated and 82 down-regulated, were identified. (**C**) Scatterplot of enriched top 30 GO pathways for DEGs. The Y-axis represented the GO pathways, and the X-axis represented the Rich factor. (**D**) Scatterplot of enriched top 20 KEGG pathways for DEGs. The Y-axis represented the KEGG pathways, and the X-axis represented the Rich factor. (**E**) Expression results of *lama3*, *lamb3*, *lamc2*, *sv2a* and *thbs1a* genes in 48 hpi zebrafish larvae. The relative change of mRNA levels was calculated by the 2 − ΔΔCt method and β-actin. Data were shown as the mean ± standard deviation (SD), and *P* values < 0.05 were considered statistically significant, *p < 0.05, **p < 0.01, ***p ≤ 0.001

In order to further explore the specific mechanism of *P. gingivalis* OMVs on cardi-ovascular damage, RNA-seq technology was employed to investigate the effects of *P. gingivalis* OMVs on the gene expression regulation in the zebrafish larvae. In this study, three biological replicates were performed in the PBS and *P. gingivalis* OMVs injected zebrafish larvae at 48 hpi, respectively. Relative to the control, 332 differentially expressed genes (DEGs), including 250 up-regulated and 82 down-regulated, were identified (Fig. 6A and B and Excel S1-S3). The GO enrichment results clarified that immune response or immune system process were the most affected pathways after *P. gingivalis* OMV injection (Fig. 6C), in which cxcl18a.1, cxcl18b, ccl34a.4, and CR762483.1 genes were enriched. The list of enriched GO terms and corresponding genes is provided in Excel S4 and S5. In addition, five KEGG pathways including the extracellular matrix (ECM)-receptor interaction, toll-like receptor signaling pathway, AGE-RAGE signaling pathway in diabetic complications, taurine and hypotaurine metabolism, retinol metabolism were significantly enriched (Fig. 6D), and their related genes are shown in Excel S6 and S7.

Since ECM-receptor interaction was significantly enriched in KEGG, this study focused on validating the genes refer to this pathway, such as *lama3*, *lamb3*, *lamc2*, *sv2a* and *thbs1a (*Excel S7). The RT-qPCR results demonstrated upregulation of those genes, which further validated the transcriptome results (Fig. 6E).

## Discussion

The results of this study revealed that OMVs secreted by the periodontal pathogen *P. gingivalis* may interact with the ECM (*lama3, lamb3, lamc2, sv2a, and thbs1a*) and nfκb (*tnfa*, *tnfb* and *il6*) signaling pathway, thereby inflicting cardiac damage and inflammatory reactions in zebrafish. Employing RNA-Seq and RT-qPCR, we determined the impact of *P. gingivalis* OMVs on cardiovascular health, indicating potential mechanisms at the genetic level that amplify the risk of cardiovascular diseases due to the predominant pathogen, *P. gingivalis*. These findings may contribute to a deeper understanding of the intricate relationship between periodontitis and cardiovascular disorders.

Given its impressive genetic congruence with humans, the zebrafish serves as an ideal specimen for these types of explorations, streamlining research methodologies and optimizing cost-efficiency [16]. Based on the results of cardiac phenotypes, we selected 2.5 µg/µL *P. gingivalis* OMVs group due to its pronounced induction of severe pericardial edema compared to other groups. Produced during the growth of *P. gingivalis*, OMVs contain numerous virulence factors, including LPS, pili, *Porphyromonas gingivalis* peptidylarginine deiminase (PPAD), gingipains and non-coding small RNA (sRNA) and could penetrate deeper tissues than the parent bacteria [25]. Characterized by a distinctive structure laden with potent virulence factors, OMVs boast a slim external membrane barrier [11]. This feature facilitated their dissemination to areas beyond the reach of their originating bacteria, amplifying their deleterious impact not only within the oral environment but also further afield [17]. Such characteristics hint at the potential process by which oral microbes could trigger remote cardiac inflammation. This intimates the mechanism through which oral microbiota might induce distal cardiac inflammatory reactions [26]. In this study, we observed that relevant cardiac phenotypes in zebrafish that microinjected different concentrations of *P. gingivalis* OMVs, in which directly induced damage to the heart, leading to pericardial enlargement, vascular damage, and simultaneously inciting inflammatory responses. Previous research has confirmed the adverse effects of *P. gingivalis* OMVs on the cardiovascular system, especially in increasing vascular permeability [17]. Our findings can be attributed to the ability of OMVs to disrupt human vascular endothelial cells (ECs) via gingipain enzymatic activity. This disruption enhances vascular permeability, resulting in heightened tissue exudate and subsequent tissue edema [27].

Regarding immune and inflammatory responses induced by *P. gingivalis* OMVs, we observed a significantly higher number of neutrophils, which were viewed as bystanders or biomarkers of cardiovascular disease [28], in the *P. gingivalis* OMV treated group. What’s more, RT-qPCR results demonstrated the upregulated expression of related inflammatory genes such as *tnfa*, *tnfb* and *il6*, which indicated the activation of nfκb signaling pathway. This finding was consistent with previous investigations. Several studies suggested the potential role of nfκb signaling pathway in activating genes involved in various cardiovascular diseases [29–32]. Mengru et al. demonstrated that *P. gingivalis* accelerated atherosclerosis via the nfκb signaling pathway [31]. It was reported that nfκb signaling pathway regulated many processes in the cardiovascular system, including inflammation, cell survival, differentiation and proliferation [33]. Pro-inflammatory cytokines and microbial products induced nfκb signaling, resulting in transcriptional regulation of pro-inflammatory genes, including cytokines, chemokines and adhesion molecules, which promoted the inflammatory process [33]. Chemokines were therapeutic targets in CVD and served as an important bridge between immune cells and inflammatory response [34]. In this certain disease setting, the nfκb pathway might be understimulated, leading to alterations in signaling and consequently gene expression, contributing to disease pathology [33]. GO analysis indicated that genes such as cxcl18a.1, cxcl18b, ccl34a.4, and CR762483.1 were enriched. These genes have been reported as a family of small, secreted, and structurally related cytokines with chemotactic properties and a crucial role in inflammation and immunity [35]. Meanwhile, they were the key regulators of recruitment and adhesion of leukocytes to inflamed arteries in the setting of atherosclerosis and into the myocardium of the ischemic heart. Therefore, we speculated that *P. gingivalis* OMVs may promote activation of nfκb signaling pathway and finally upregulates chemokine genes expression such as cxcl18a.1, cxcl18b, ccl34a.4, and CR762483.1, inducing host immune response and causing distal cardiovascular injury. Further gene overexpression and knockdown experiments are needed to validate the above conjectures.

Previous investigations demonstrated that ECM-receptor interaction signaling pathway exhibited a vital role in development, wound healing and regeneration [36–38]. In the present study, the result of KEGG enrichment analysis showed that zebrafish exposed to *P. gingivalis* OMVs exhibited a marked activation of the ECM receptor interaction signaling pathway. This finding was in accordance with other studies that highlighted the critical role of ECM in cardiovascular growth, sustenance, and restoration [39–42]. Through its influence on the typical scar tissue repair process, the ECM structure promotes angiogenesis and tissue growth, particularly in post-myocardial infarction [43–45]. Served as ligands for cell receptors or regulating its architecture, ECMs could influence cell activities such as adhesion, migration, proliferation, apoptosis, survival and differentiation [37]. Tetrachlorobisphenol A (TCBPA) was shown to interfere with nervous and cardiovascular development through this pathway in zebrafish embryos through transcriptomic analysis [46]. And 3,3’-di-O-methylellagic acid 4’-glucoside (DMAG) was found to be accelerated platelet recovery and megakaryopoiesis by activating this pathway [47]. Dysregulation of this pathway can lead to development and progression of diseases such as cancer and cardiovascular disease [37]. One study detected that ECM-related genes (*lamb3* and *lama3*) upregulated in pancreatic cancer and influenced tumor progression and prognosis, validating the significant contribution of ECM to the pathophysiological process [48]. In this study, RT-qPCR results showed that the expression of five ECM-related genes were upregulated in the *P.gingivalis* OMVs treated groups, which was consistent with the results of previous studies [48, 49]. Further experiments are needed to explore the upstream regulatory factors of this pathway.

Despite the meticulous design of the experiment, there are still some limitations to this study. Firstly, periodontitis etiology hinges on the ecological imbalance between the host and oral microbiota [6]. The tissue destruction resulting from periodontitis is an exacerbated inflammatory reaction of the host against the parasitic periodontal pathogens in dental plaque. Among these pathogens, *P. gingivalis* remains a predominant subject of oral research. While our study was centered around *P. gingivalis*, the pathogenic mechanisms of other bacteria and their potential synergistic interactions necessitate further exploration. Moreover, it is imperative that we undertake comprehensive validation studies on both cellular and protein aspects to solidify our understanding of the link between OMVs and cardiovascular diseases. Upcoming research should include pathway inhibitors, ensuring a retrospective validation of the pathway’s dependability. Furthermore, future investigations should be more targeted, focusing specifically on conditions like infective endocarditis. The creation of specialized inhibitors holds promise as potential treatments for persistent forms of endocarditis, whether idiopathic or refractory. Effectively managing periodontal tissue infections may also play a pivotal role in halting the advancement of cardiovascular ailments. This study observed that the predominant pathogens linked with periodontitis had the potential to inflict vascular injuries. Motivated by these findings, we delved into a comprehensive exploration of associated pathways and underlying mechanisms, aiming to unravel the genesis of this interconnection. Our ultimate objective is to reinforce the foundation of clinical research, enabling a more nuanced understanding of periodontal challenges faced by cardiovascular patients and, in turn, enriching the preventive and therapeutic strategies against cardiovascular ailments.

## Conclusions

Within the limitations of this study, the conclusions were drawn as follows:

1. *P. gingivalis* OMVs have been implicated in pericardial distension, vascular impairments, and, to a certain degree, a heightened susceptibility to cardiovascular diseases. They predominantly trigger the host’s immune defense, as evidenced by the proliferation of neutrophils, the activation of the nfκb signaling cascade, and the amplification of inflammatory mediators.
2. Delving into transcriptomics, we discerned the involvement of the immune response and the ECM-receptor interaction signaling pathways in this intricate process. This segment of our investigation offers pivotal insights into the ramifications of periodontitis on cardiovascular diseases, paving the way for advanced research and prophylaxis of the associated mechanisms.

### Electronic supplementary material

Below is the link to the electronic supplementary material.

Supplementary Material 1

Supplementary Material 2

Supplementary Material 3

Supplementary Material 4

Supplementary Material 5

Supplementary Material 6

Supplementary Material 7

Supplementary Material 8

## Data Availability

The datasets generated and analyzed during the current study are available in the NCBI repository https://www.ncbi.nlm.nih.gov/bioproject/PRJNA1022139/, accession number PRJNA1022139.

## Abbreviations

OMVs: Outer membrane vesicles
*P. gingivalis*: *Porphyromonas gingivalis*
CVD: Cardiovascular disease
WT: Wild-type
ECM: Extracellular matrix
LPS: Lipopolysaccharides
TEM: Transmission electron microscope
NSA: Nanoparticle size analyzer
