# Supplementary material for: Effects and mechanisms of Porphyromonas gingivalis outer membrane vesicles induced cardiovascular injury
Source: BMC Oral Health. 2024 Jan 19;24:112. doi: 10.1186/s12903-024-03886-7 (PMC10799447; doi:10.1186/s12903-024-03886-7)
Supplement: Supplementary file 1 — Supplementary Material 1 [file 12903_2024_3886_MOESM1_ESM.docx]

**Table S1.** Primers used for RT-qPCR.

| **Primer name** | **Direction (5'-3')** |
| --- | --- |
| *β-actin*-RT-F | TCTCTTGCTCCTTCCACCAT |
| *β-actin*-RT-R | TGCTTGCTGATCCACATCTG |
| *gata4*-RT-F | GGCATTCAGACACGAAAGCG |
| *gata4*-RT-R | TGGTTCAGTCTTGATGGGTCG |
| *nkx2.5*-RT-F | ACACCTACCCTGCGTTTAGT |
| *nkx2.5*-RT-R | TGAAGGAACACTGCTGGATTG |
| *cdh5*-RT-F | GCCTGTCACGATAGCAGCTT |
| *cdh5*-RT-R | GATCACGATCTGCCTCGTCC |
| *Tnfa*-RT-F | ATCAGCTGCACGTCTGAACT |
| *tnfa*-RT-F | GCAGATTGAGCGGATTGCAC |
| *tnfb*-RT-F | GTACCTGAGCCACACCATCA |
| *tnfb*-RT-F | GGCTCCAAGGTAAATGGTGC |
| *il6*-RT-F | CGGTCCACTCGATCCTGTTC |
| *il6*-RT-F | TCCTCTTGGGGTCTTTCCCT |
| *lama3*-RT-F | GTGACTACACTCCAGCCGAC |
| *lama3*-RT-F | GCACGCTTCATGGTGATGTC |
| *lamb3*-RT-F | CAGGACGCACTGTAAATGATGC |
| *lamb3*-RT-F | CGTTAATGCCGTGTCTCTTGG |
| *lamc2*-RT-F | TCTTGAGCAATCTAACAGCCATT |
| *lamc2*-RT-F | CCATCATATTCAGCAGGACATCT |
| *sv2a*-RT-F | CTGACCACCATGCCTGAGAG |
| *sv2a*-RT-F | GGCTTCTCCACCCATGTCAA |
| *thbs1a*-RT-F | GCGGTCGAGGGATTCAACAG |
| *thbs1a*-RT-F | CACTCTTGCGTGTAGCAGTCT |
